# Survival of castration‐resistant prostate cancer patients treated with dendritic–tumor cell hybridomas is negatively correlated with changes in peripheral blood CD56^bright^CD16^−^ natural killer cells

**DOI:** 10.1002/ctm2.505

**Published:** 2021-08-26

**Authors:** Helena Haque Chowdhury, Simon Hawlina, Mateja Gabrijel, Saša Trkov Bobnar, Marko Kreft, Gordan Lenart, Marko Cukjati, Andreja Nataša Kopitar, Nataša Kejžar, Alojz Ihan, Luka Ležaič, Marko Grmek, Andrej Kmetec, Matjaž Jeras, Robert Zorec

**Affiliations:** ^1^ Laboratory of Cell Engineering Celica Biomedical Ljubljana Slovenia; ^2^ Laboratory of Neuroendocrinology – Molecular Cell Physiology, Institute of Pathophysiology, Faculty of Medicine University of Ljubljana Ljubljana Slovenia; ^3^ Clinical Department of Urology University Medical Centre Ljubljana Ljubljana Slovenia; ^4^ Department of Surgery, Faculty of Medicine University of Ljubljana Ljubljana Slovenia; ^5^ CPAE, Department of Biology, Biotechnical Faculty University of Ljubljana Ljubljana Slovenia; ^6^ Blood Transfusion Centre of Slovenia Ljubljana Slovenia; ^7^ Institute of Microbiology and Immunology, Faculty of Medicine University of Ljubljana Ljubljana Slovenia; ^8^ Institute for Biostatistics and Medical Informatics, Faculty of Medicine University of Ljubljana Ljubljana Slovenia; ^9^ Department of Nuclear Medicine University Medical Centre Ljubljana Ljubljana Slovenia; ^10^ Department of Radiology, Faculty of Medicine University of Ljubljana Ljubljana Slovenia; ^11^ Faculty of Pharmacy University of Ljubljana Ljubljana Slovenia

Dear Editor,

We investigated the clinical outcome of treating castration‐resistant prostate cancer (CRPC) patients with autologous immunohybridoma cell (aHyC) vaccine generated by electrofusing autologous dendritic (DC) and tumor cells (TC), and tested whether the immunological response, involving the CD56^bright^CD16^−^ natural killer (NK), putative pro‐metastatic cells,^1^, ^2^ correlates with survival of CRPC patients. The results demonstrated that aHyC treatment is safe and prolongs patient survival correlating with a decrease in peripheral blood CD56^bright^CD16^−^ NK cells.

Despite advances in cancer immunotherapy, the only approved CRPC immunotherapy to date is a cell‐based vaccine (sipuleucel‐T),^3^ with a single antigen‐specific response induction mechanism, consisting of a small fraction of DC markers. DCs are able to activate both naive and memory T cells, ideally suited for augmenting antitumor immune responses.^4^ Consistent with this, vaccination with enriched blood‐derived DCs loaded with three tumor‐associated antigens resulted in more frequent detection of antigen‐specific T cells in CRPC patients.^5^

Here, whole TCs were electrofused with DCs to produce aHyC vaccine.^6^ The advantage of such hybridomas is their capacity of presenting both known and yet unknown tumor‐associated antigens to T‐lymphocytes. We used aHyC vaccine to treat chemotherapy‐naive CRPC patients in a phase 1/2 randomized, placebo‐controlled crossover trial to test primary outcomes—feasibility, safety, and quality of life (QL)—and also to evaluate clinical and immunological outcomes with overall survival (OS).

Twenty‐two men with CRPC were included (Table S1, Figure S1); 19 of them were treated with all four doses of the aHyC vaccine, either in first (aHyC‐first group, n = 12) or in the second (placebo‐first group, n = 10) trial session. Both groups were balanced with respect to most of the other considered variables (Table S2).

The treatment with aHyC revealed only a few and mild (grade 1) intervention‐related adverse events (AEs; Figure 1A–C), and did not cause additional or more frequent AEs than placebo, indicating that recorded AEs were not directly related to the aHyC application. None of the patients required hospitalization. Renal and liver functions remained stable during and after the aHyC treatment. These results show that the treatment of CRPC patients with aHyC is feasible and safe.

**FIGURE 1:**
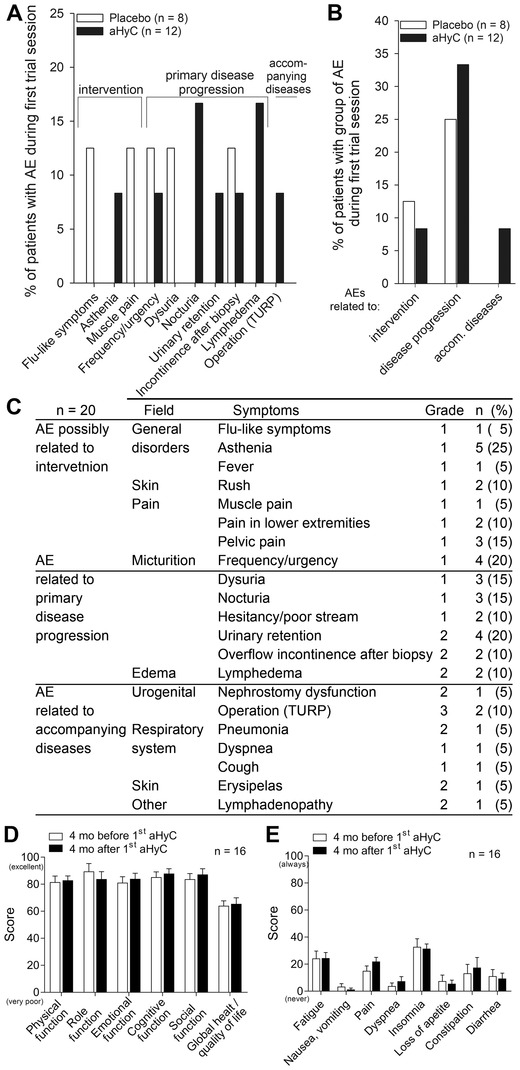
Adverse events and quality of life overview. Comparison of frequencies (%) of recorded adverse events (AEs) (A) and grouped AEs (B) between the autologous hybridoma cell group (aHyC; *n* = 12) and the placebo group (*n* = 8) during the first trial session until the crossover (about 17‐week period) showed no differences between placebo and aHyC groups (Fisher exact test). The frequencies of AEs related to intervention (*P* = 0.65), primary disease (*P *= 0.92), and accompanying disease (*P* = 0.83) were all similar between groups. (C) Data of all recorded AEs collected from all patients (*n* = 20) during the applications and 6 months after the last application (aHyC vaccine or placebo, respectively) showed that only few patients had treatment‐related adverse events, those were all mild, grade 1. The most frequent AE was asthenia (five patients; 25%), followed by frequency/urgency (four patients; 20%) and urinary retention (four patients; 20%). For statistical analysis, a *z*‐test for proportions and the Fisher exact test were used. Graphical presentations of quality of life and functionality (D) and symptoms (E) assessed from the EORTC QLQ‐C30 questionnaire show high scores of functionality and a low burden of symptoms that remained unchanged during aHyC treatment. For each patient, the data collected from all questionnaires in the period before the first autologous hybridoma cell (aHyC) vaccine application were averaged, and the same was done for the data acquired during/after the treatment (twice per patient). Data in the histograms are given as mean scores of 16 patients before (white columns) versus mean scores of the same patients after (black columns) the first aHyC vaccine treatment, with SEM (error bars). The Student's paired *t*‐test was used. No statistically significant differences were detected following aHyC vaccine treatment. aHyC, autologous hybridoma cell; mo, months; TURP, transurethral resection of the prostate

QL was unchanged with aHyC treatment (QL scored 64.0 ± 3.7 before vs. 65.5 ± 4.5 after the first aHyC treatment; *P *= 0.67). Different modes of functioning, all scoring above 80 (Figure 1D), and various symptoms (scoring below 40; Figure 1E) were also comparable before and after treatment, indicating that the aHyC treatment did not affect the patients’ overall wellbeing. The demonstrated safety/nontoxicity is consistent with the completely autologous nature of aHyC.

The baseline median prostate‐specific antigen (PSA) value was higher in the aHyC group (8.9 ng/ml; interquartile range [IQR] = 5.6–23.7 ng/ml) than in the placebo‐first group (4.3 ng/ml; IQR = 3.9–7.7 ng/ml; Figures 2 and S3), as reported.^7^ The median PSA progression time (PSA‐P) and median PSA doubling time (PSA‐DT) from first aHyC/placebo application (Table S2) were not significantly different between the two groups. High‐sensitivity CRP, an inflammatory marker, was higher in the aHyC‐first group (Figure 2B), which correlates with the kinetics of the PSA values (Figure 2A). In trials with DC vaccines, as well as in this study, there was no correlation between survival and PSA levels measured at different time points (not shown), likely due to the relatively delayed clinical response after immunotherapy compared with cytotoxic therapy.^8^

**FIGURE 2:**
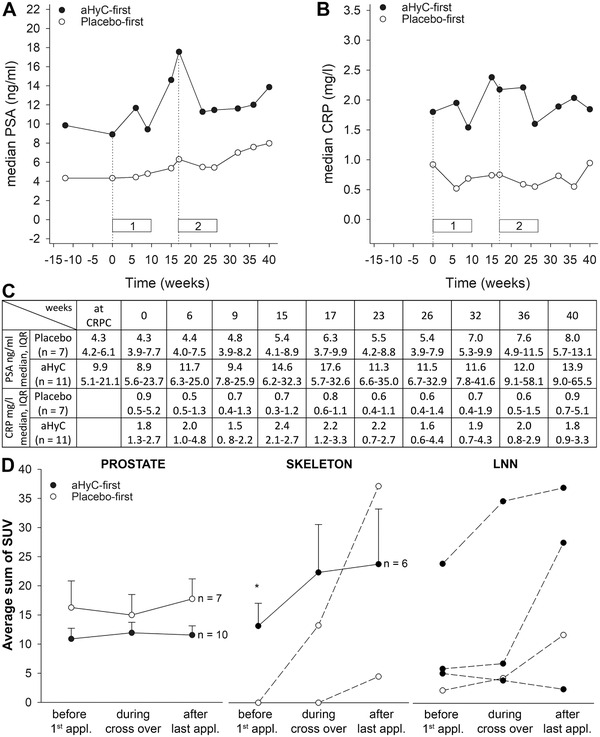
The analysis of prostate‐specific antigen (PSA), C‐reactive protein (CRP), and [^18^F]fluorocholine PET–CT lesions. Graphical presentation of clinical parameters: PSA (A) and CRP (B) over the trial period, with numerical values (C). Following aHyC treatment, PSA decreased transiently in nine patients and permanently in one patient, but median serum PSA levels determined at several time points increased after aHyC and placebo. Time points compare median values in each group, including at the time of CRPC diagnosis (12 weeks), at the baseline (at first application, time 0), just before the crossover (17 weeks), around the time of the last application (23 weeks), and 4 months after it (40 weeks). In each group, one patient was excluded from this analysis (death and chemotherapy). Interquartile ranges are omitted for clarity but are given in Table  (C). Dotted vertical lines denote the time of first applications: at the beginning of the trial and at the crossover; horizontal bars denote the length of the first (1) and second (2) application rounds. (D) Average (solid lines) and individual (dashed lines, for *n* < 5) standardized uptake values (SUVs) of lesions visualized by [^18^F]fluorocholine PET–CT in prostate, skeleton, and lymph nodes (LNN) shown at three measured time points: just before the first application of placebo or aHyC, at the time of crossover, and after concluding the second round of treatment in the crossover phase. Data were analyzed only in patients with lesions in a certain region. Error bars are SEM and are shown only in the positive direction for transparency. Paired and unpaired Student's *t*‐tests were used, as appropriate. The asterisk denotes a statistically significant difference between both groups (*P* < 0.05)

The standardized uptake values (SUVs) of [^18^F]fluorocholine PET–CT scans showed improvements in individual patients after treatment with aHyC. In the aHyC group, a continuous decrease in SUV was observed in the prostate (two patients; Figure S4) and in the lymph nodes and skeleton (one patient), and a transient SUV decrease in the prostate (six patients) and in the skeleton (two patients). In the placebo‐first group, the SUV decreased transiently in four patients. There were no significant differences in the average SUVs between the two groups (Figure 2D). The SUV appears to have stabilized in the prostate and skeleton 6 months after the first aHyC treatment, but not in the lymph nodes (Figure 2D).

Peripheral blood leukocytes were monitored regularly during the trial (Table 1). At baseline, the levels of all cell populations were similar between the two groups. After the first trial session, the total CD3^+^ T cells increased in both groups. However, an increase in regulatory CD25^++^CD127^low^, activated helper CD4^+^CD69^+^, and cytotoxic T cells (CD8^+^) and a decrease in total NK cells compared to baseline were recorded only in aHyC‐first group (Table 1). Between treatment groups, a significant change was observed only in CD56^bright^CD16^−^ NK cells, the level of which was significantly lower in the aHyC‐ versus the placebo‐treated patients (*P *= 0.04; Figure 3A,B). Human NK lymphocytes are involved in antitumor immunity, and CD56^bright^CD16^−^ NK cells are considered immunoregulatory cytokine‐producing cells, representing 5%–10% of all NK cells in peripheral blood.^9^ The levels of counterpart CD56^dim^CD16^+^ NK cells were unaltered compared to baseline in both groups. These results indicate that the application of aHyC affects the immune system through NK cell subpopulation, consistent with observations in other cancers.^1^, ^2^

**TABLE 1:** Peripheral blood leukocyte populations monitored during the trial

	Placebo‐first (*n* = 8)		aHyC‐first (*n* = 12)		*P* (placebo vs. aHyC)	
	Baseline (%)	After application (%)	Baseline (%)	After application (%)	Baseline	After application
Total CD3^+^ T cells^a^	73.9 ± 3.1	77.0 ± 2.5*	74.8 ± 2.4	76.7 ± 2.2**	0.81	0.93
Total CD4^+^ T cells^b^	64.0 ± 3.4	62.7 ± 3.2	65.6 ± 5.4	64.1 ± 5.4	0.82	0.85
CD25^++^CD127^low^ (Treg)^d^	7.0 ± 0.8	7.6 ± 0.6	7.1 ± 0.6	8.3 ± 0.6**	0.94	0.44
CD4^+^CD69^+^ ^b^	5.3 ± 0.6	7.7 ± 1.0	5.3 ± 0.7	7.4 ± 0.9**	0.95	0.79
CD4^+^CD152^+^ ^b^	6.9 ± 2.2	5.7 ± 1.2	7.3 ± 2.5	4.6 ± 0.6	0.91	0.37
Total CD8^+^ T cells^b^	31.1 ± 3.5	32.0 ± 2.7	30.5 ± 4.9	32.0 ± 5.0	0.93	1.00
CD8^+^CD69^+^ ^b^	3.1 ± 0.8	4.3 ± 1.1	3.7 ± 0.9	3.2 ± 0.6	0.66	0.40
CD8^+^CD152^+^ ^b^	2.1 ± 0.3	2.6 ± 0.8	2.2 ± 0.5	1.5 ± 0.2	0.86	0.21
Total NK cells^a^	18.2 ± 2.5	15.8 ± 2.0	16.5 ± 1.8	15.3 ± 1.6*	0.57	0.85
CD56d^im^CD16^+ ^ ^c^	87.9 ± 2.0	83.9 ± 2.5	89.2 ± 1.7	87.9 ± 1.6	0.63	0.17
CD56b^right^CD16^−^ ^c^	4.9 ± 0.9	8.1 ± 1.3**	4.5 ± 0.7	5.2 ± 0.6	0.75	0.04*
Total NKT cells^a^	9.1 ± 1.7	9.7 ± 1.6	9.4 ± 2.5	11.8 ± 2.6	0.92	0.57
CD25^+^CD4^+^ ^a^	15.1 ± 0.9	16.0 ± 1.0	19.1 ± 2.8	18.0 ± 2.3	0.20	0.45
CD19^+^ (B cells)^a^	7.6 ± 0.9	7.1 ± 1.0	8.3 ± 1.1	7.7 ± 1.0	0.68	0.69
CD4^+^ T cells^a^	47.1 ± 2.7	48.1 ± 2.4	48.5 ± 3.8	48.5 ± 3.8	0.79	0.93
CD8^+^ T cells^a^	23.1 ± 3.0	24.8 ± 2.5	23.4 ± 4.3	25.1 ± 4.5*	0.96	0.95

*Note*: After application: after completing the firstapplication round, that is, four applications of aHyC or placebo; bold values: significant differences compared to baseline values or between the two groups. *P < 0.05, **P < 0.01 compared to baseline (paired *t*‐test) or between groups (Student's *t*‐test).

^a^ Percentage of cells relative to all lymphocytes.

^b^ Percentage of cells relative to CD3^+^ cells.

^c^ Percentage of cells per all NK cells.

^d^ Percentage of cells relative to all CD4^+^ cells.

**FIGURE 3:**
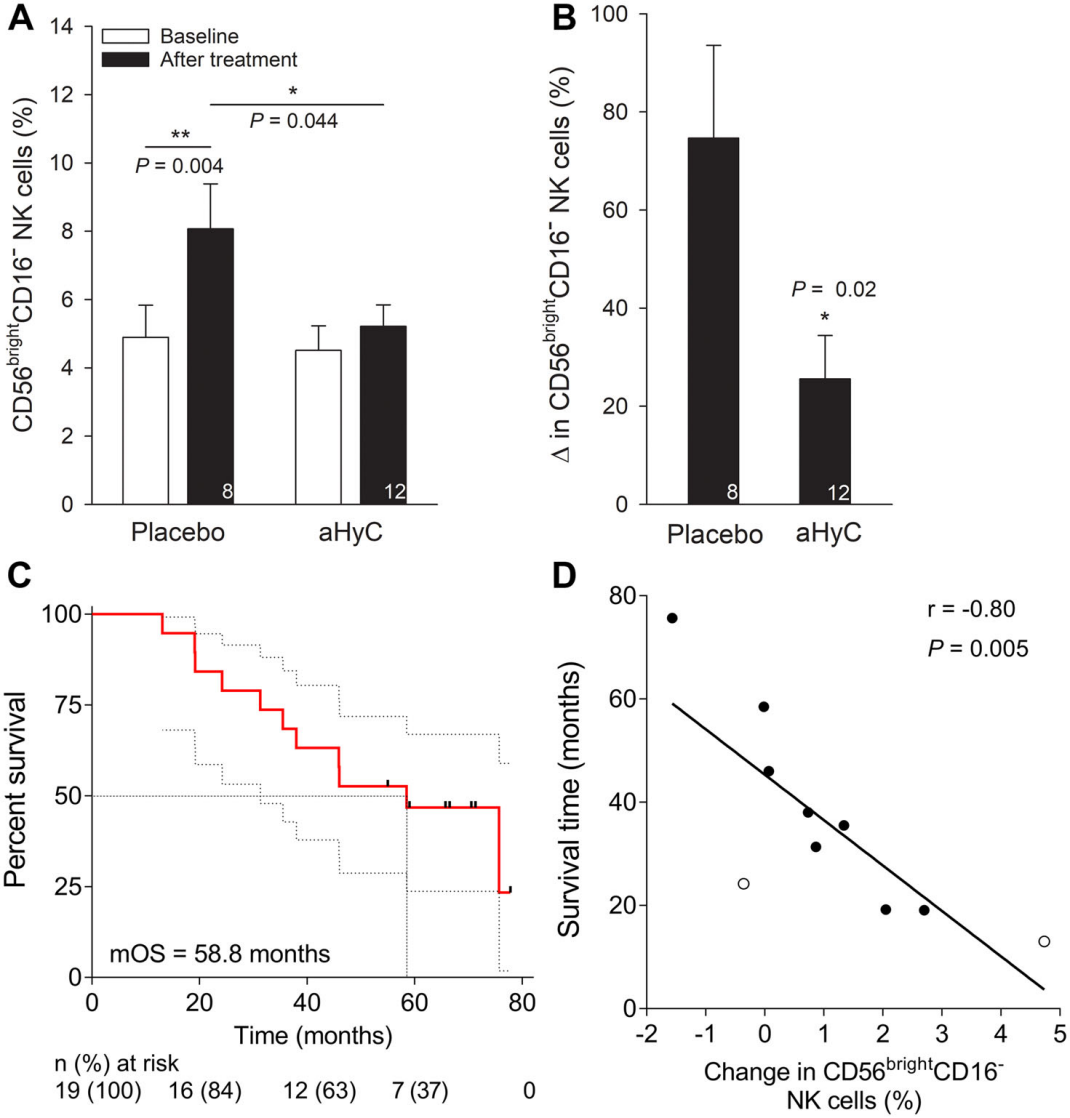
Survival of aHyC‐treated patients correlates with changes in peripheral blood CD56^bright^CD16^−^ natural killer cells. During the trial, the CD56^bright^CD16^−^ subpopulation of natural killer (NK) cells was measured in the blood samples as the percentage of all NK cells. (A) Changes in the percentage of the CD56^bright^CD16^−^ NK cell subpopulation in the placebo‐first (left columns) and in the aHyC‐first groups (right columns) at baseline (open columns; time period of up to 4 months before the first application) and before the crossover (black columns). Note that the CD56^bright^CD16^−^ fraction of NK cells significantly increased only in the placebo‐first group compared to baseline. (B) Relative change (%) in CD56^bright^CD16^−^ NK cell subpopulation relative to baseline after placebo and aHyC application, respectively. Bars represent mean values with SEM, and the numbers of patients are shown within the bars. Paired and unpaired Student's *t*‐tests were used, as appropriate, *P < 0.05. (C) Kaplan–Meier plot of overall survival (OS) with 95% confidence interval (gray curves) in all patients who were treated with aHyC (*n* = 19). Survival time was measured from the first application of aHyC   until cutt‐off date or death. Black symbols denote censored subjects; dotted lines denote median value, *n* (%) denotes the numbers of patients at risk. (D) The correlation between survival time of deceased patients (*n* = 10; from PCa—black symbols, other causes—open symbols) and changes in percentages of CD56^bright^CD16^−^ NK cells relative to baseline, determined after the crossover. *r* represents Pearson correlation coefficient. The regression line is of the form: Survival time [months] = (–8.8 ± 0.3 [months/%]) × change in CD56^bright^CD16^−^ NK cells [%] + (45.3 ± 4.6 [months])

Survival analysis included all patients who received all four doses of aHyC vaccine (*n* = 19) and was determined from the first application of aHyC to the cutoff date or the patient's death (any cause). The median OS was 58.5 months (95% confidence interval [CI], 38.8–78.2; Figure 3C). The incidence of any cause of death was 58% (11 patients). Cancer‐specific survival was 75.7 months (95% CI, 41.1–110.4). Compared to previous publication,^5^ aHyC treatment demonstrated to be beneficial for patient survival, especially since seven patients (37 %) were initially diagnosed with a less responsive, metastatic disease.

Negative correlation between the survival time and change in the CD56^bright^CD16^−^ fraction of NK cells at the end of the trial (Figure 3D, *r* = –0.80, 95% CI, –0.95 to –0.34, *P *= 0.005) suggests that a relatively high increase in peripheral CD56^bright^CD16^−^ NK cells shortens survival. Similarly, a negative correlation between the abundance of CD56^bright^CD16^−^ NK cells and OS in melanoma patients was observed.^10^

In conclusion, these results indicate that aHyC treatment attenuates an increase in CD56^bright^CD16^−^ NK cell subpopulation in peripheral blood, benefiting CRPC patient survival.

## ETHICS APPROVAL AND CONSENT TO PARTICIPATE

This study was conducted in accordance with the provisions of the Declaration of Helsinki and was approved in June 2013 by the National Medical Ethics Committee and the Agency for Medicinal Products and Medical Devices of the Republic of Slovenia, part of European Medical Agency (EMA). Trial EMA registration: EUDRACT: 2012‐005498‐29. All participants signed written informed consent prior to inclusion in the study.

## CONFLICT OF INTEREST

The authors declare no conflict of interest.

## FUNDING INFORMATION

This work was supported by grants P3 310, J3 6790, J3 6789, and J3 9266 from the Slovenian Research Agency, by CipKeBip, COST Action BM1002, EU COST Action CM1207‐GLISTEN, and EU COST Action CA 15214 EuroCellNet. The funding sources had no involvement in study design, collection, analysis and interpretation of data, the writing of the report, and the decision to submit the article for publication.

## AUTHOR CONTRIBUTIONS

H.H.C., S.H., M. Gabrijel, M.K., A.I., M.J., and R.Z. conceptualized the study. H.H.C., M. Gabrijel, S.T.B., M.C., and M.J. contributed in methodology. H.H.C., M. Gabrijel, and S.T.B. helped in validation. H.H.C., M.K., and N.K. helped in formal analysis. H.H.C., S.H., M. Gabrijel, S.T.B., A.N.K., L.L., and M. Grmek investigated the study. H.H.C., S.H., A.N.K., L.L., and M. Grmek contributed in data curation. H.H.C. and S.H. wrote the original draft. All the authors reviewed and edited the manuscript. H.H.C. and R.Z. directed the study. H.H.C., S.H., M. Gabrijel, A.I., A.K., and R.Z. supervised the project. S.H., G.L., A.I., and A.K. provided resources. S.H., A.I., and R.Z. acquired funding. N.K. provided software.

## DATA AVAILABILITY STATEMENT

Data generated and analyzed during the current study are available from the corresponding author on reasonable request. Clinical trial protocol is available at link: lnmcp.mf.uni‐lj.si/Protocol.pdf. Contact Matjaž Jeras for the immunology part.

## Supporting information

Supporting Information

## References

[1] Mamessier E, Pradel LC, Thibult ML, et al. Peripheral blood NK cells from breast cancer patients are tumor‐induced composite subsets. J Immunol. 2013;190(5):2424‐2436. 23359508 10.4049/jimmunol.1200140

[2] Holtan SG, Creedon DJ, Thompson MA, Nevala WK, Markovic SN. Expansion of CD16‐negative natural killer cells in the peripheral blood of patients with metastatic melanoma. Clin Dev Immunol. 2011;2011:316314. 21403861 10.1155/2011/316314 PMC3049347

[3] Kantoff PW, Higano CS, Shore ND, et al. Sipuleucel‐T immunotherapy for castration‐resistant prostate cancer. N Engl J Med. 2010;363(5):411‐422. 20818862 10.1056/NEJMoa1001294

[4] Koido S. Dendritic‐tumor fusion cell‐based cancer vaccines. Int J Mol Sci. 2016;17(6):828. 10.3390/ijms17060828 PMC4926362 27240347

[5] Westdorp H, Creemers JHA, van Oort IM, et al. Blood‐derived dendritic cell vaccinations induce immune responses that correlate with clinical outcome in patients with chemo‐naive castration‐resistant prostate cancer. J Immunother Cancer. 2019;7(1):302. 31727154 10.1186/s40425-019-0787-6 PMC6854814

[6] Gabrijel M, Kreft M, Zorec R. Monitoring lysosomal fusion in electrofused hybridoma cells. Biochim Biophys Acta. 2008;1778(2):483‐490. 17996722 10.1016/j.bbamem.2007.10.013

[7] Waeckerle‐Men Y, Uetz‐von Allmen E, Fopp M, et al. Dendritic cell‐based multi‐epitope immunotherapy of hormone‐refractory prostate carcinoma. Cancer Immunol Immunother. 2006;55(12):1524‐1533. 16612599 10.1007/s00262-006-0157-3 PMC11031118

[8] Shore ND. Advances in the understanding of cancer immunotherapy. BJU Int. 2015;116(3):321‐329. 24612369 10.1111/bju.12692

[9] Michel T, Poli A, Cuapio A, et al. Human CD56^bright^ NK cells: an update. J Immunol. 2016;196(7):2923‐2931. 26994304 10.4049/jimmunol.1502570

[10] de Jonge K, Ebering A, Nassiri S, et al. Circulating CD56(bright) NK cells inversely correlate with survival of melanoma patients. Sci Rep. 2019;9(1):4487. 30872676 10.1038/s41598-019-40933-8 PMC6418246

