# Supplementary material for: Survival of castration‐resistant prostate cancer patients treated with dendritic–tumor cell hybridomas is negatively correlated with changes in peripheral blood CD56brightCD16− natural killer cells
Source: Clin Transl Med. 2021 Aug 26;11(8):e505. doi: 10.1002/ctm2.505 (PMC8387785; doi:10.1002/ctm2.505)
Supplement: Supplementary file 1 — Supporting Information [file CTM2-11-e505-s001.docx]

###### Letter-to-Editor (Manuscript ID CTM2-2021-04-0743.R1)

SUPPLEMENTARY MATERIAL

**Survival of castration-resistant prostate cancer patients treated with dendritic-tumor cell hybridomas is negatively correlated with changes in peripheral blood CD56^bright^CD16^−^ natural killer cells**

Helena Haque Chowdhury^1,2†^, Simon Hawlina^3,4†^, Mateja Gabrijel^1,2^, Saša Trkov Bobnar^1,2^, Marko Kreft^1,2,5^, Gordan Lenart^3^, Marko Cukjati^6^, Andreja Nataša Kopitar^7^, Nataša Kejžar^8^, Alojz Ihan^7^, Luka Ležaič^9,10^, Marko Grmek^9^, Andrej Kmetec^3^, Matjaž Jeras^1,11,#^, Robert Zorec^1,2^*

^1^Laboratory of Cell Engineering, Celica Biomedical, Ljubljana, Slovenia.

^2^Laboratory of Neuroendocrinology – Molecular Cell Physiology, Institute of Pathophysiology, Faculty of Medicine, University of Ljubljana, Ljubljana, Slovenia.

^3^Clinical Department of Urology, University Medical Centre Ljubljana, Ljubljana, Slovenia. ^4^Department of Surgery, Faculty of Medicine, University of Ljubljana, Ljubljana, Slovenia.

^5^CPAE, Department of Biology, Biotechnical Faculty, University of Ljubljana, Ljubljana, Slovenia.

^6^Blood Transfusion Centre of Slovenia, Ljubljana, Slovenia.

^7^Institute of Microbiology and Immunology, Faculty of Medicine, University of Ljubljana, Ljubljana, Slovenia.

^8^Institute for Biostatistics and Medical Informatics, Faculty of Medicine, University of Ljubljana, Ljubljana, Slovenia.

^9^Department of Nuclear Medicine, University Medical Centre Ljubljana, Ljubljana, Slovenia.

^10^Department of Radiology, Faculty of Medicine, University of Ljubljana, Ljubljana, Slovenia.

^11^Faculty of Pharmacy, University of Ljubljana, Ljubljana, Slovenia.

†Contributed equally to this article [helena.chowdhury@mf.uni-lj.si](mailto:helena.chowdhury@mf.uni-lj.si); [simon.hawlina@kclj.si](mailto:simon.hawlina@kclj.si). #Contact for immunology part [matjaz.jeras@ffa.uni-lj.si](mailto:matjaz.jeras@ffa.uni-lj.si). * Correspondence: [robert.zorec@mf.uni-lj.si](mailto:robert.zorec@mf.uni-lj.si).

**Running title:** Immunohybridomas Treat Prostate Cancer

**Materials and Methods**

*Experimental design and patients*

This was a single-institution (Urology Department, University Medical Centre Ljubljana), randomized, double-blind, placebo-controlled cross-over phase 1/2 clinical trial of autologous hybridoma cell (aHyC) vaccine treatment vs. placebo (vehicle), conducted from 2013 to 2016, to evaluate feasibility, safety and QL, as well as to evaluate clinical and immune outcomes and overall survival (OS). We used electrofusion of autologous prostate tumor cells and immature monocyte-derived DCs to produce the aHyC vaccine, which was injected back into the patient to induce antitumor responses. Electrofusion resulted in fusion of the plasmalemma and lysosomes within hybridomas, exhibiting *in vitro* T-cell-induced cytotoxicity against tumor cells.[1, 2]

This study was conducted in accordance with the provisions of the Declaration of Helsinki and was approved in June 2013 by the National Medical Ethics Committee and the Agency for Medicinal Products and Medical Devices of the Republic of Slovenia, part of European Medical Agency (EMA). Trial EMA registration: EUDRACT: 2012-005498-29.[3] All participants signed written informed consent prior to inclusion in the study.

**Patients in the Trial**

Patients were consecutively recruited and treated from February 2014 until August 2016 and were followed up for survival until September 2020. Among other inclusion/exclusion criteria (Table S1), eligible patients were all chemotherapy naive, asymptomatic or minimally symptomatic adult men with a biopsy-confirmed diagnosis of PCa and disease progression to CRPC without visceral metastases. They did not receive any other antitumor therapy before or during the trial, with the exception of androgen-deprivation therapy (but not enzalutamide, abiraterone acetate or newer anti-androgens) and the standard supportive therapy for prevention of skeleton-related events (i.e., denosumab, once monthly).[4] The two most common reasons for ineligibility of patients for the trial were that their disease had not yet progressed to CRPC (65%) and that they were symptomatic. If symptoms related to disease progression or radiological progression assessed by positron emission tomography-computed tomography (PET-CT) [^18^F]fluorocholine in patients included in the trial were observed during the trial, routine examinations (i.e. nuclear bone scintigraphy, CT of abdomen/thorax) were performed, and the patient was presented to an oncologist for further established CRPC next in-line therapy (abiraterone acetate, enzalutamide, docetaxel, Ra 223). This therapy ended his participation in the study.

Eligible patients had to respond to tetanus toxoid and diphtheria (Td-pur) subcutaneous injection with a positive cutaneous delayed-type hypersensitivity (DTH) reaction to provide readout of their immune system responsiveness. All patients responded positively to the DTH test, with an average local skin reaction diameter of 4.4 ± 0.4 cm. Patients were then randomized into an active (aHyC) and a placebo group; basic patient characteristics of both groups are described in Table S2. Before the first application of either aHyC or placebo (vehicle of the aHyC vaccine), all participants in both groups were preconditioned with low-dose metronomic cyclophosphamide to attenuate regulatory T cells (Treg),[5] which downregulate antigen-specific immune responses.[6] Because the allogeneic reaction consists of a strong pro-inflammatory immune response of the recipient's T cells directly recognizing non-self MHC molecules present on allogeneic transplants,[7, 8] all patients intravenously received an irradiated (25 Gy) allogeneic buffy coat, freshly prepared from 450 mL ABO, Kell and Rh, compatible whole blood unit, concomitantly with each aHyC and placebo application to non-specifically rouse up their immune status.

Each patient received four injections of aHyC vaccine or placebo at 3-week intervals. After a 7-week cross-over interval, members of both groups crossed over to another group, yielding a total of 32 weeks of time on trial.

**Randomization and Masking**

Based on the annual incidence of new patients with CRPC in the country (about 120/2 million), we anticipated inclusion of up to 25 participants. A random allocation sequence for 26 patients in a 1:1 ratio (aHyC/placebo) was computer generated by a block randomization method, printed and sealed in opaque envelopes and handed to the production staff. Twenty-two patients were enrolled in the study by invitation of the key clinical researcher and were consecutively assigned an enrollment number (1–22). They were then directed to the intervention, receiving either placebo-first (n = 10) or aHyC-first (n = 12) according to the randomized allocation sequence that matched the patient’s enrollment sequence number.

For double blinding, the appearance of the syringes containing placebo or aHyC was identical and masked with coded labeling of the vaccine and its recipient. Un-blinding was only done at the examiner’s request, e.g. if the primary disease worsened and the patient had to discontinue the trial (3 patients). Final un-blinding was made at the end of the clinical trial after the last visit of the last patient.

**Trial Objectives**

Primary

- Feasibility and safety. Records of adverse events (AEs) during the trial and the background medical history data were collected. AEs were categorized into three groups related to intervention, primary disease progression and accompanying disease, standardized according to the Common Terminology Criteria for Adverse Events (CTCAE v5.0).
- Quality of life assessment.[9, 10] European Organization for Research and Treatment of Cancer Core Quality of Life Questionnaire (EORTC QLQ-C30) was completed at each patient’s visit.

Secondary

- Type and extent of immune and clinical responses to the treatment.[11, 12] Serum prostate-specific antigen (PSA), C-reactive protein (CRP), and levels of peripheral blood lymphocytes were measured during the trial; DTH skin reactions were assessed, and [^18^F]fluorocholine PET-CT-choline scans were analyzed.
- Overall survival (OS). For all patients who received aHyC vaccine (n = 19), median OS (mOS, in months) was analyzed from the first application of aHyC.

*Method details*

**Vaccine Preparation**

Autologous Tumor Cells

In an outpatient facility, 25–35 prostate biopsy samples per patient were taken under local anesthesia (10 mL of 2% xylocaine periprostatic injection, plus 10 mL of 2% xylocaine jelly intrarectal; AstraZeneca) using transrectal ultrasonography (B-K Medical, UltraView 800) and a biopsy gun with 18G needles (Magnum; Bard Biopsy Systems). The removal of prostate tumor biopsies did not result in substantial bleeding, infection or in other side effects or inconvenience to the patients. All patients received oral Ciprofloxacin prophylaxis (2 × 500 mg daily for 5 days) and were advised to refrain from physical activities. Two to three biopsy samples were used for histopathological examination to confirm the presence of tumor cells; from the rest, tumor cells (TCs) were isolated by mechanical and enzyme (collagenase, 1 mg/mL; Gibco) dissociation, after which suspension of TCs was γ-irradiated (300 Gy), frozen in four aliquots and kept in liquid nitrogen until further use.

Autologous Monocyte-Derived Dendritic Cells

The patients’ white blood cells were collected with leukapheresis at the Blood Transfusion Centre of Slovenia. At the vaccine production facility autologous monocytes were isolated by adhesion on a plastic surface and differentiated into nonadherent immature DCs in a defined medium containing GMP-grade rhGM-CSF and rhIL-4 (Miltenyi Biotec).[13] Samples were frozen in four aliquots for further use.

Investigational Product Preparation

Autologous TCs and autologous immature DCs were thawed and washed. DCs were pre-incubated in a defined medium containing rhGM-CSF. Both, TCs and DCs were washed, resuspended in a fusion medium (5% glucose) and fused by electrostimulation in a custom-made chamber.[14] After 24 h incubation with DC-maturation factors,[15, 16] the cells were washed and resuspended in Ringer’s lactate solution, which was also used as a vehicle for the aHyC vaccine (placebo). The median number of viable cells in each vaccine was 7.7 × 10^6^ (ranging from 2.2 to 14.4 × 10^6^), with 3–10% of hybridomas. Criteria for investigational product release: the number of cells ≥ 1 × 10^6^, cell viability > 50% and the radiation dose report confirmation. The DC maturation was evaluated with flow cytometry (Figure S2). The vaccine was packed into a syringe with a 21G needle, delivered to the outpatient facility and administered subcutaneously in the lower inner part of the right femoral triangle.

**Serum Markers**

Each patient's blood samples were collected before the trial and then before each aHyC/placebo and/or denosumab application. Serum levels of PSA and CRP were monitored during the trial (on average 20 times per patient). PSA progression (PSA-P) time was defined as the time from the start of treatment to the date of the first PSA test result that represented a 25% or greater increase from the PSA nadir and an absolute increase of at least 2 ng/mL above nadir, confirmed by another PSA value at least 3 weeks later.[17] PSA doubling time (PSA–DT) was calculated using PSA values from the first application (aHyC or placebo), according to the formula: [log2]/[slope coefficient from regressing the log of PSA over time].

**DTH Testing**

For DTH testing during the trial, autologous lysed TCs (10^5^ tumor cells/100 μL) were injected subdermally either into the upper back or into the left or right lower arm at the time of each application of the aHyC/placebo vaccine, with the exception of patients in the placebo-first group who received only placebo-DTH in the first period. TCs were lysed by three consecutive freeze-thaw cycles and subsequently centrifuged. A suspension of KLH (keyhole limpet hemocyanin; 50 μg, Stellar Biotechnologies) was first used as an immunogen (T cell-dependent antigen) and then as a positive control throughout the trial, while Ringer’s lactate solution was used as a negative DTH control. Both were injected subdermally near the site of TC lysate application. All DTH reactions to lysed autologous tumor cells in all patients were negative, likely due to the limited quantity of lysed tumor cells injected (scarcity of tumor biopsy material) and because antitumor immune responses were not yet fully established at the time of the DTH assays, a characteristic of immune therapies.[16] Reactions to KLH were all positive.

**Flow Cytometry to Evaluate the Peripheral Blood Leukocytes**

During the clinical trial, blood samples of participating patients were collected (17 times per patient on average) to monitor the fractions of various leukocyte populations (total NK cells, CD16^+^CD56^dim^ and CD16^−^CD56^bright^ NK subpopulations; total NKT cells; total CD3^+^ T cells; total CD4^+^ T cells, CD4^+^CD69^+^ and CD4^+^CD152^+^ populations of T cells; fractions of CD4^+^ and CD8^+^ relative to lymphocytes, total CD8^+^ T cells, CD8^+^CD69^+^ and CD8^+^CD152^+^ populations of T cells; CD25^+^CD4^+^; CD25^++^CD127^low^ Treg cells; and CD19^+^ B cells). Changes in the percentages of cell subpopulations in patients during the trial were analyzed by comparing the calculated average percentage of cells up to 4 months before the first placebo or aHyC vaccine application with the average values after the first application until the time point of cross-over. The values were then analyzed and compared between both groups (placebo and aHyC). Leukocyte populations and DCs were analyzed on the flow cytometer (BD Canto II, BD Biosciences) using DIVA software (BD Biosciences).

We used the following antibodies: anti-CD69 FITC, anti-CD152 PE, anti-CD8 PerCP Cy5.5, anti-CD3 APC, anti-CD4 APC Cy7, anti-CD25 FITC, anti-CD127 PE, anti-CD4 PerCP Cy5.5, anti-CD3 FITC, anti-CD16 PE, anti-CD56 APC, anti-HLA-DR FITC, anti-CD83 PE, anti-CD86 PE-Cy7 and anti-CD80 APC. All antibodies were obtained from BD Biosciences, except anti-CD19 FITC, CD56 PE, CD4 PE-Cy5, CD8 FITC and CD3PE (Cytognos).

Sample Preparation

One hundred microliters of peripheral blood were incubated with 20 µL of antibodies for 20 min at room temperature in the dark. Erythrocytes were lysed by incubation for 10 min with BD FACSTM Lysing Solution (BD Biosciences). Samples were centrifuged for 5 min at 450 × g and subsequently washed twice with cold phosphate-buffered saline (PBS). Cell pellets were then resuspended in 1 mL of PBS. A total of 30,000 events were recorded.

Cell Population Abundance

The expression of cell antigens was measured on lymphocytes from fresh whole blood collected into EDTA Vacutainer tubes. Autologous monocyte-derived DCs were prepared from the patients’ white blood cells collected by leukapheresis at the Blood Transfusion Centre of Slovenia and then transported to the vaccine manufacturing facility. The maturation and activation of DCs was assessed according to expression of CD80, CD86, CD83, and HLA-DR. A total of 30,000 events were measured.

Gating Strategy

Peripheral blood mononuclear cells (PBMCs) were identified based on the side scatter (SSC) and forward scatter (FSC). Lymphocytes were also positioned based on their SSC and FSC. B lymphocytes were identified from lymphocytes based on CD19 expression, and NK cells based on CD56 and CD16 expression. The latter were divided into two populations: CD56^bright^ cells and CD56^dim^ CD16^+/−^ cells. Subpopulations of T lymphocytes were identified based on CD3 expression as CD4^+^ T helper cells, expressing CD69 or CD152, and CD8^+^ cytotoxic T cells, which express CD69 or CD152. Regulatory T cells were gated on CD4^+^ cells expressing CD25^high^ and CD127^low^. The maturation and activation markers (HLA-DR, CD80, CD86 and CD83) were measured on DCs.

###### [^18^F]fluorocholine PET-CT

[^18^F]-fluorocholine PET-CT imaging of the abdomen and chest (from the cranial base to the middle of the thigh) was performed before starting the treatment protocol, allowing the starting (baseline) numbers of all measurable target lesions to be determined. Each scan was divided into four parts: prostate, skeleton, lymph nodes and parenchyma to calculate the sums of the standardized uptake values (SUVs) of up to the five largest target lesions (basic comparative values) in each part of the scan and then to compare them with the same SUV sums calculated from further [^18^F]fluorocholine PET-CT scans performed during the cross-over and after the treatment. Only those patients who continued to the cross-over phase of the trial and thus received a placebo and aHyC regimens (n = 17), were analyzed for changes in SUVs in prostate, lymph nodes and skeleton. Data on [^18^F]fluorocholine PET-CT lesions were analyzed using the Student’s t-test (paired, where appropriate) and with analysis of variance. No parenchymal SUVs were found in the aHyC group.

**Data Processing and Statistical Analysis**

For the safety evaluation of the aHyC intervention, we predicted that less than 50% of patients (49%) would experience one or more mild to moderate AE in the aHyC-group and none in the placebo-group. A sample size of 11 patients per group, with a 25% drop-out rate is sufficient to detect this difference using a two-tailed z-test of proportions between two groups with 80% power and a 5% level of significance.

During the trial, two of the 22 patients were excluded, one before the treatment (additional malignancy) and the other one after (non-adherence). Additionally, two patients in the aHyC-group and one patient in the placebo-group concluded the trial at the cross-over because of disease progression. Nineteen patients received all four doses of aHyC.

The overall safety evaluation period was set at 6 months before the first application and 6 months after the last application of aHyC or placebo, and up to 4 months before and 4 months after the first application of the aHyC vaccine for the assessment of QL. For the comparison of AEs between aHyC and placebo, the observation period was set from the first application in the first period until the start of cross-over and for the analysis, a z-test for proportions and the Fisher exact test were used. For QL analysis, a paired t-test was used. For the assessment of QL and overall symptoms and functioning, patients were asked to complete the standard EORTC QLQ-C30 questionnaire[18] every time they visited the outpatient facility during the trial, and data were analyzed as described.[19] The median number of forms collected in 4 months before and 4 months after the first application of aHyC vaccines was 2 and 3 per patient, respectively. Only patients who received aHyC vaccines (n = 19) and from whom requested data were collected before and after the treatment with aHyC (n = 16) were considered in the analysis. All parameters were averaged for all patients and are presented as the mean ± SEM (Figure 1).

For evaluation and comparison, median baseline prostate-specific antigen (PSA) and C-reactive protein (CRP) levels were determined only in patients who were followed up during the entire observation period (1 patient in each group was excluded from the analysis; Figure 2A-C). Models of PSA dynamics in time were done using the linear mixed-effect regression method. For peripheral blood leukocytes, paired and unpaired Student’s t-tests were used, as appropriate. For further evaluation of CD56^bright^CD16^−^ changes, the percentages of CD56^bright^CD16^−^ cells were analyzed at baseline and after the first trial session (before the cross-over). Two-sided *P* values of ≤ 0.05 were considered to indicate statistical significance.

#### The effect of aHyC treatment on survival was assessed by the log-rank test (Mantel-Cox) and the hazard ratio (HR) with a 95% confidence interval (CI) based on the Mantel-Haenszel regression model and presented on Kaplan-Meier plots. For the correlation analysis between the survival time and absolute change in the CD56^bright^CD16^−^ fraction of NK cells, recorded at the end of the trial relative to baseline, data of 10 out of 11 deceased patients was analyzed, for whom the afore-mentioned data were available. Analyses were performed using Excel (Microsoft), Sigma Plot (Systat Software SigmaPlot, RRID:SCR_003210), GraphPad Prism (GraphPad Software, RRID:SCR_002798), R statistical programs[20] and gofLMM (gofLMM Version 0.0.1 statistical package for R. Goodness of fit for linear mixed-effect models).

**Table S1. Inclusion/Exclusion Criteria for the Selection of Patients**

| Characteristics of the disease | Histologically proven prostate cancer | | | |
| --- | --- | --- | --- | --- |
|  | Progressive disease: confirmed by three consecutive increases in PSA values measured successively at least 2-week intervals, despite castration testosterone levels (<50 ng/dL) and PSA >2 ng/mL | | | |
| Parallel therapy: before starting the treatment and at the beginning of the treatment | No signs of previous therapy toxicity | | | |
|  | Immune therapies | | No previous application of autologous or allogeneic antitumor vaccines | |
|  |  |  | No previous or current immunotherapy | |
|  | Endocrine evaluation/therapy | | Patients should be without corticosteroid or any other therapy of adrenal insufficiency for at least 2 weeks before vaccination | |
|  |  |  | Hormonal therapy permitted to maintain castration levels of testosterone | |
|  | Chemotherapy naive | | Before and during the trial, patients should not receive chemotherapy, radiological treatment, enzalutamide or abiraterone acetate | |
| Characteristics of patients | Men must be 18 years of age and older. They must be informed about the research nature of the trial and sign written informed consent. | | | |
|  | Capacity status: Karnofsky 70%–100% | | | |
|  | Expected survival: at least 12 months^A^ | | | |
|  | Blood tests: determined twice (within 45 days and 72 h before entering the trial) | | | Leukocytes >3.8 × 10^9^/L  Lymphocytes >500 × 10^9^/L  Platelets >120 × 10^9^/L  Hemoglobin >100 g/L |
|  | Blood group (ABO, Kell, RhD) and HLA phenotype determination | | | |
|  | Liver function | Bilirubin <20 μmol/L | | |
|  | Renal function | Creatinine <120 μmol/L | | |
|  | Lung function | No serious lung disease | | |
|  | Cardiovascular function | NYHA I/II status | | |
|  |  | No angina pectoris, clinically significant cardiac arrhythmias or recent (6 months) heart attacks | | |
|  | Immunologic evaluation | A positive DTH response to standard historical antigens (tetanus and diphtheria toxoids); induction at the application site after 48 h with a diameter of more than 5 mm | | |
|  | Radiographic evaluation of disease | [^18^F]fluorocholine PET-CT up to 4 weeks before entering the trial | | |
| Other | - No active infectious disease requiring antibiotic or antiviral therapy  - No severe pain requiring chronic opioid analgesia  - No HIV, HBV, or HCV infection (anti-HIV, HbsAg, and Hep C; viral nucleic acid test negative)  - No serious medically unmanageable disease  - No use of metformin^B^  - No parallel secondary malignant diseases  - No previous extensive lymph node irradiation  - No vasculitis and other autoimmune diseases  - No alcohol addiction or dependence on other intoxicating substances  - No psychiatric illnesses that could affect the trial  - No use of hydroxyurea within 45 days before the beginning of the trial  - No use of immunomodulators within 30 days before the beginning of the trial  - No previous prostate radiotherapy^B^  - No previous radical prostate surgery^B^ | | | |

PSA, prostate-specific antigen; NYHA, New York heart Association; DTH, delayed-type hypersensitivity; PET, positron emission tomography; CT, computed tomography; HIV, human immunodeficiency virus; HBV, hepatitis B virus; HCV, hepatitis C virus. ^A^Patients should have Eastern Cooperative Oncology Group performance status from 0 to 1 (<https://ecog-acrin.org/resources/ecog-performance-status>). ^B^Criteria acceptable to include patients in the documented control group.

**Table S2. Baseline Patient Characteristics**

|  | Treatment group | |
| --- | --- | --- |
|  | aHyC | Placebo |
| Number | 12 | 8 |
| Age (years), median (IQR) at first application | 75.0 (72.1–80.2) | 74.1 (66.3–81.0) |
| PSA (ng/mL), median (IQR) |  |  |
| At PCa diagnosis | 42.5 (21.3–72.0) | 35.0 (24.8–40.8) |
| At first application | 8.4 (5.7–19.9) | 5.8 (4.1–11.1) |
| Gleason score at PCa diagnosis, n (%) |  |  |
| 8–10 | 10 (83) | 7 (88) |
| 6–7 | 2 (17) | 1 (13) |
| Metastases at first application according to [^18^F]fluorocholine PET-CT |  |  |
| No metastases | 5 (42) | 7 (87) |
| Oligometastases (≤3) | 3 (25) | 0 (0) |
| Polymetastases (≥4) | 4 (33) | 1 (13) |
| Site of metastases*, n (%) |  |  |
| Bone | 4 (33) | 0 |
| Lymph node | 0 | 0 |
| Bone + lymph node | 3 (25) | 1 (13) |
| Visceral | 0 | 0 |
| Time from PCa diagnosis to first application (years), median (IQR) | 4.6 (3.2–5.3) | 5.0 (4.2–5.5) |
| After the trial: |  |  |
| Next-in-line treatment (docetaxel, enzalutamide, abiraterone acetate), n (%) | 8 (67) | 6 (75) |
| Deaths after last application, n (%) |  |  |
| PCa | 6 (50) | 4 (50) |
| Within 1 year | 0 | 1 |
| Within 2 years | 0 | 2 |
| >2 years | 6 | 1 |
| Non-PCa | 2 | 0 |
| PSA-P (months), median (IQR), *P* | 3.0 (2.1–7.1) | 4.5 (3.7–14.6), *P* = 0.06 |
| PSA-DT (months), median (IQR), *P* | 5.6 (1.9–33.7) | 8.8 (2.7–9.4), *P* = 0.91 |

aHyC, autologous hybridoma cell; IQR, interquartile range; PCa, prostate cancer; PSA, prostate-specific antigen; PSA-P, PSA-progression time; PSA-DT, PSA-doubling time.

Excluded (n = 110)

Inclusion criteria not met (n = 109)

Refused to participate (n = 1)

Assessed for eligibility (n = 132)

Allocation
(n = 22)

Randomly assigned to aHyC (n = 12)

aHyC received (n = 12)

aHyC not received (n = 0)

Disease progression (n = 0)

Death (n = 0)

Other (n = 0)

Randomly assigned to placebo (n = 10)

Placebo received (n = 9)

Placebo not received (n = 1)

Disease progression (n = 0)

Death (n = 0)

Other (n = 1; additional malignancy)

Placebo received (n = 10)

Placebo not received (n = 2)

Disease progression (n = 2)

Death (n = 0)

aHyC received (n = 8)

aHyC not received (n = 1)

Disease progression (n = 0)

Death (n = 1)

Lost to follow-up (n = 0)

Discontinued (n = 0)

Lost to follow-up (n = 0)

Discontinued (n = 0)

Continue to
cross-over
(n = 21)

Follow

-up

Block randomization (n = 26)

Analyzed for OS (n = 12)

Excluded from OS analysis (n = 0)

Analyzed for OS (n = 7)

Excluded from OS analysis (n = 1; not received aHyC)

Analysis of OS

(n = 19)

Analyzed (n = 12)

Excluded from analysis (n = 0)

Analyzed (n = 8)

Excluded from analysis (n = 1; non-adherence to the protocol)

Analysis

(n = 20)

## Figure S1. Patient Selection

A total of 22 patients were recruited from February 2014 to January 2016. aHyC, autologous hybridoma cell; OS, overall survival.





## Figure S2. Maturation of Dendritic Cells

The ratio of dendritic cell (DC) maturation markers, measured by flow cytometry, between monocyte-derived DCs (immature DCs [iDCs]) and DCs after *in vitro* maturation (mature DCs [mDCs]). Bars are means of all 19 patients who received autologous hybridoma cell vaccines; error bars are standard error of the mean. Asterisks denote significant difference from the ratio of 1 (n = 19; **P* < 0.05; ** *P* < 0.01; *** *P* < 0.001), indicating a change in DC phenotype toward maturation.





## **Figure S3. Analysis of the Prostate-Specific Antigen (PSA)**

(**A**) Fixed-effect fit for the linear mixed-effect regression model of PSA over time (500 to 36 days before the first application): Twenty patients had PSA measured from 2 to 8 times. The log-transformed PSA model was adjusted for random intercept and slope for each patient. The goodness of fit test showed no lack of fit (gofLMM Version 0.0.1 statistical package for R; available upon request from rokblagus@mf.uni-lj.si). (Log)PSA in patients with CRPC increased significantly over time (positive regression coefficient of 0.005, *P* < 0.001). The grey band denotes the 95% confidence interval.
(**B**) Fixed-effect fit for the linear mixed-effect regression model of PSA over time (500 days before the first application until the 110^th^ day after the point of cross-over): twenty patients had more than seven measurements of PSA. Non-linear splines for time before treatment and interaction between the time-patient groups were allowed for in the log-transformed PSA model. This was further adjusted for a random intercept and slope for each patient. The goodness of fit test showed no lack of fit. Significant (log)PSA effect over time before treatment is evident; however, there are no statistically significant effects over time after treatment and/or due to the group of the patients. The reason for non-significant effects over time after treatment might be partly due to the relatively short time interval of 110 days. Grey intensity bands denote 95% confidence intervals for the two groups. aHyC, autologous hybridoma cell vaccine.

**
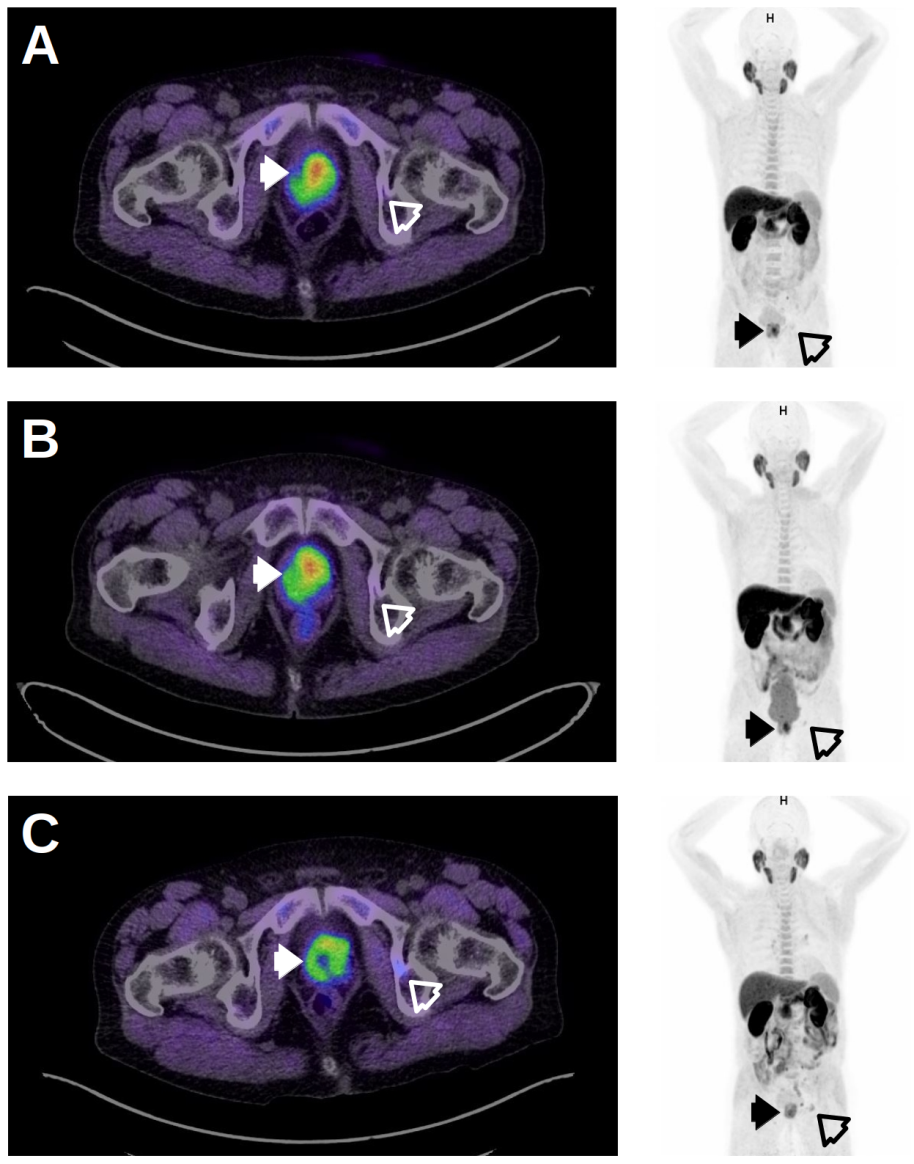
**

**Figure S4. Response assessment by [^18^F]fluorocholine PET-CT.**

Fusion images ([^18^F]fluorocholine PET and CT image combined, colour image, left panels, colour scale range blue → green → yellow → red signifies increasing intensity of uptake) and whole-body maximum intensity projection (MIP) images (monochrome, right panels, dark colour signifies increasing intensity of uptake) of a patient at baseline (**A**) and two follow-up examinations (**B**, **C**) during the trial. At baseline PET-CT examination (**A**), intense uptake of the radiopharmaceutical in the prostate is observed, most intensively in the anterior part (A, full arrows). Three months later, after the therapy with aHyC vaccine (at cross-over), there is a slight decrease in the uptake of the radiopharmaceutical (**B**, full arrows), which is more prominent at the end of the trial (**C**, full arrows). The visual decrease in the uptake of radiotracer is reflected also by quantitative index (standardized uptake values, SUVs: baseline scan – 10.8 (A), first follow-up: 10.4 (B), second follow-up: 8.0 (C)). Please note the uptake of the radiopharmaceutical into the skeletal lesion in the left ischiadic bone (empty arrow), which appeared to increase gradually from A to C (SUVs: baseline scan – 0.0 (A), first follow-up: 2.7 (B), second follow-up: 4.7(C)), demonstrating the ability of [^18^F]fluorocholine PET-CT to detect and assess subtle divergent changes in cancer lesions during therapy.

## **References**

[1] M. Gabrijel, M. Kreft, R. Zorec, Monitoring lysosomal fusion in electrofused hybridoma cells, Biochimica et biophysica acta, 1778 (2008) 483-490.

[2] M. Gabrijel, M. Bergant, M. Kreft, M. Jeras, R. Zorec, Fused late endocytic compartments and immunostimulatory capacity of dendritic-tumor cell hybridomas, The Journal of membrane biology, 229 (2009) 11-18.

[3] E.C.T. Register, <https://www.clinicaltrialsregister.eu/ctr-search/trial/2012-005498-29/SI>.

[4] M.R. Smith, F. Saad, R. Coleman, N. Shore, K. Fizazi, B. Tombal, K. Miller, P. Sieber, L. Karsh, R. Damiao, T.L. Tammela, B. Egerdie, H. Van Poppel, J. Chin, J. Morote, F. Gomez-Veiga, T. Borkowski, Z. Ye, A. Kupic, R. Dansey, C. Goessl, Denosumab and bone-metastasis-free survival in men with castration-resistant prostate cancer: results of a phase 3, randomised, placebo-controlled trial, Lancet (London, England), 379 (2012) 39-46.

[5] L.E. Noerregaard, E. Ellebaek, T. Zeeberg, Influence of Metronomic Cyclophosphamide and Interleukine-2 alone or Combined on Blood Regulatory T Cells in Patients with Advanced Malignant Melanoma Treated with Dendritic Cell Vaccines, Journal of Clinical & Cellular Immunology, 03 (2012).

[6] D.T. Le, E.M. Jaffee, Regulatory T-cell modulation using cyclophosphamide in vaccine approaches: a current perspective, Cancer research, 72 (2012) 3439-3444.

[7] J.W. Fabre, The allogeneic response and tumor immunity, Nature medicine, 7 (2001) 649-652.

[8] D. Laurin, J. Kanitakis, J. Bienvenu, C. Bardin, J. Bernaud, S. Lebecque, L. Gebuhrer, D. Rigal, A. Eljaafari, Allogeneic reaction induces dendritic cell maturation through proinflammatory cytokine secretion, Transplantation, 77 (2004) 267-275.

[9] C.U. Avelino, R.M. Cardoso, S.S. Aguiar, M.J. Silva, Assessment of quality of life in patients with advanced non-small cell lung carcinoma treated with a combination of carboplatin and paclitaxel, Jornal brasileiro de pneumologia : publicacao oficial da Sociedade Brasileira de Pneumologia e Tisilogia, 41 (2015) 133-142.

[10] R. Sosnowski, M. Kulpa, M. Kosowicz, F. Presicce, F. Porpiglia, A. Tubaro, D.E.N. C, T. Demkow, Basic methods for the assessment of health-related quality of life in uro-oncological patients, Minerva urologica e nefrologica = The Italian journal of urology and nephrology, 69 (2017) 409-420.

[11] M.O. Frank, J. Kaufman, S. Tian, M. Suarez-Farinas, S. Parveen, N.E. Blachere, M.J. Morris, S. Slovin, H.I. Scher, M.L. Albert, R.B. Darnell, Harnessing naturally occurring tumor immunity: a clinical vaccine trial in prostate cancer, PloS one, 5 (2010).

[12] A. Hoos, A.M. Eggermont, S. Janetzki, F.S. Hodi, R. Ibrahim, A. Anderson, R. Humphrey, B. Blumenstein, L. Old, J. Wolchok, Improved endpoints for cancer immunotherapy trials, Journal of the National Cancer Institute, 102 (2010) 1388-1397.

[13] S. Nair, G.E. Archer, T.F. Tedder, Isolation and generation of human dendritic cells, Current protocols in immunology, Chapter 7 (2012) Unit7.32.

[14] R. Zorec, M. Kreft, M. Gabrijel, Method for determining the quantity and quality of hybridomas, Munich: European Patent Office, (2010).

[15] J.J. Lee, K.A. Foon, R.B. Mailliard, R. Muthuswamy, P. Kalinski, Type 1-polarized dendritic cells loaded with autologous tumor are a potent immunogen against chronic lymphocytic leukemia, Journal of leukocyte biology, 84 (2008) 319-325.

[16] A. ten Brinke, G. van Schijndel, R. Visser, T.D. de Gruijl, J.J. Zwaginga, S.M. van Ham, Monophosphoryl lipid A plus IFNgamma maturation of dendritic cells induces antigen-specific CD8+ cytotoxic T cells with high cytolytic potential, Cancer immunology, immunotherapy : CII, 59 (2010) 1185-1195.

[17] M. Hussain, B. Goldman, C. Tangen, C.S. Higano, D.P. Petrylak, G. Wilding, A.M. Akdas, E.J. Small, B.J. Donnelly, S.K. Sundram, P.A. Burch, R.S. Dipaola, E.D. Crawford, Prostate-specific antigen progression predicts overall survival in patients with metastatic prostate cancer: data from Southwest Oncology Group Trials 9346 (Intergroup Study 0162) and 9916, J Clin Oncol, 27 (2009) 2450-2456.

[18] P. Fayers, Aaronson, N. K., Bjordal, K., Groenvold, M., Curran, D., & Bottomley, A. , EORTC QLQ-C30 Scoring Manual. (3rd ed.), Brussels: European Organisation for Research and Treatment of Cancer., (2001).

[19] N.K. Aaronson, S. Ahmedzai, B. Bergman, M. Bullinger, A. Cull, N.J. Duez, A. Filiberti, H. Flechtner, S.B. Fleishman, J.C. de Haes, et al., The European Organization for Research and Treatment of Cancer QLQ-C30: a quality-of-life instrument for use in international clinical trials in oncology, Journal of the National Cancer Institute, 85 (1993) 365-376.

[20] R.C. Team, R: A language and environment for statistical computing, R Foundation for Statistical Computing, Vienna, Austria. URL <https://www.R-project.org/>. 2019.
